# In vivo MRI of the human finger at 7 T

**DOI:** 10.1002/mrm.26645

**Published:** 2017-03-10

**Authors:** Elmar Laistler, Barbara Dymerska, Jürgen Sieg, Sigrun Goluch, Roberta Frass‐Kriegl, Andre Kuehne, Ewald Moser

**Affiliations:** ^1^ Center for Medical Physics and Biomedical Engineering Medical University of Vienna Vienna Austria; ^2^ High Field MR Center Medical University of Vienna Vienna Austria; ^3^ Department of Biomedical Imaging and Image‐guided Therapy Medical University of Vienna Vienna Austria; ^4^ MRI.TOOLS GmbH Berlin Germany

**Keywords:** ultrahigh field, MRI, solenoid, RF coil, MR microscopy, human finger, 3D visualization, tendons, ligaments, vasculature, Pacinian corpuscle

## Abstract

**Purpose:**

To demonstrate a dedicated setup for ultrahigh resolution MR imaging of the human finger in vivo.

**Methods:**

A radiofrequency coil was designed for optimized signal homogeneity and sensitivity in the finger at ultrahigh magnetic field strength (7 T), providing high measurement sensitivity. Imaging sequences (2D turbo‐spin echo (TSE) and 3D magnetization‐prepared rapid acquisition gradient echo (MPRAGE)) were adapted for high spatial resolution and good contrast of different tissues in the finger, while keeping acquisition time below 10 minutes. Data was postprocessed to display finger structures in three dimensions.

**Results:**

3D MPRAGE data with isotropic resolution of 200 µm, along with 2D TSE images with in‐plane resolutions of 58 × 78 µm^2^ and 100 × 97 µm^2^, allowed clear identification of various anatomical features such as bone and bone marrow, tendons and annular ligaments, cartilage, arteries and veins, nerves, and Pacinian corpuscles.

**Conclusion:**

Using this dedicated finger coil at 7 T, together with adapted acquisition sequences, it is possible to depict the internal structures of the human finger in vivo within patient‐compatible measurement time. It may serve as a tool for diagnosis and treatment monitoring in pathologies ranging from inflammatory or erosive joint diseases to injuries of tendons and ligaments to nervous or vascular disorders in the finger. Magn Reson Med 79:588–592, 2018. © 2017 The Authors Magnetic Resonance in Medicine published by Wiley Periodicals, Inc. on behalf of International Society for Magnetic Resonance in Medicine. This is an open access article under the terms of the Creative Commons Attribution License, which permits use, distribution and reproduction in any medium, provided the original work is properly cited.

## INTRODUCTION

The most commonly used modality for imaging of the finger is standard radiography 1, with the limitation of lacking 3D information. Furthermore, ultrasound and MRI 2 are employed.

High‐resolution image data can facilitate early detection and monitoring of erosive changes 3 or inflammation 4, 5 of finger joints. Moreover, ligament and tendon injuries in the finger, common in athletes 6, have been studied using MRI at 0.35 T 7, or both 1.5T‐MRI and ultrasound 8. Other structures and pathologies of the finger also have been investigated, for example, the skin 9, finger tumors 10, vascular structure 11, and blood flow velocity 12. Few MRI experiments showed nervous structures, in particular Pacinian corpuscles in vivo in the fingertip at 1.5 T 13 and in the toe at 3 T 14. In a cadaver study, 76 µm in‐plane MRI images of the fingertip acquired in a small‐bore 7T‐scanner have been demonstrated and correlated with histology 15.

Here, we describe a method to improve the homogeneity of solenoidal coils by parametrization of the coil geometry and simulation of the resulting magnetic (B) fields. Subsequently, we explore the possibilities of this dedicated hardware for in vivo imaging of the human finger at ultrahigh field strength.

## METHODS

### Radio Frequency Coil

#### Design and Simulations

Coil length was determined by the requirement that proximal and the distal interphalangeal joints could be imaged within the field of view of the coil. By measuring the average length of the middle phalanx in 10 healthy subjects and adding a margin of 10 mm on each side, a length of 56 mm was chosen. The inner‐coil housing diameter was chosen to be 28 mm to accommodate even the largest finger in our sample (diameter 25.5 mm). The diameter of the coil windings was therefore set to 34 mm, accounting for the 3‐mm housing thickness.

At this size, a maximum of three windings was achievable to keep the self‐resonance frequency of the coil sufficiently above the Larmor frequency of 297.2 MHz.

The solenoidal coil presented has a modified geometry to optimize signal homogeneity along its axis by making the windings denser at the edges and sparser in the center 16, compensating for the signal dropoff at the edges. The geometry was subdivided in three segments: a first segment with the conductor wound at zero pitch; a second segment where the pitch increased linearly; and a third segment in which the pitch was constant, as in a regular solenoid. These regions were mirrored at the coil center (Supporting Fig. S1a).

Simulations were performed to optimize the geometry of the coil for homogeneity. Due to the small size of the coil with respect to the wavelength, the much faster quasi‐static simulation approach using Biot‐Savart's law was used instead of full‐wave electromagnetic simulations. In simulation, the angular ranges for the first and second segments, *α* and *β*, were varied from 0 ° to 180 ° in a step size of 10˚, resulting in *19·19 = 361* different coil geometries. For each geometry, homogeneity as the standard deviation of the B_1_ field, and sensitivity as the average amplitude of the B_1_ field were calculated for the inner coil volume. The pair (*α, β*) representing the optimum with respect to both homogeneity and sensitivity was chosen for the final geometry.

A full‐wave 3D electromagnetic simulation using commercial software packages (XFdtd 7.3, Remcom, State College, PA, USA; ADS, Agilent, Santa Clara, CA, USA) was performed on the final geometry, including a realistic index finger phantom to determine the specific absorption rate (SAR) averaged over 10 g of tissue.

**Figure 1:**
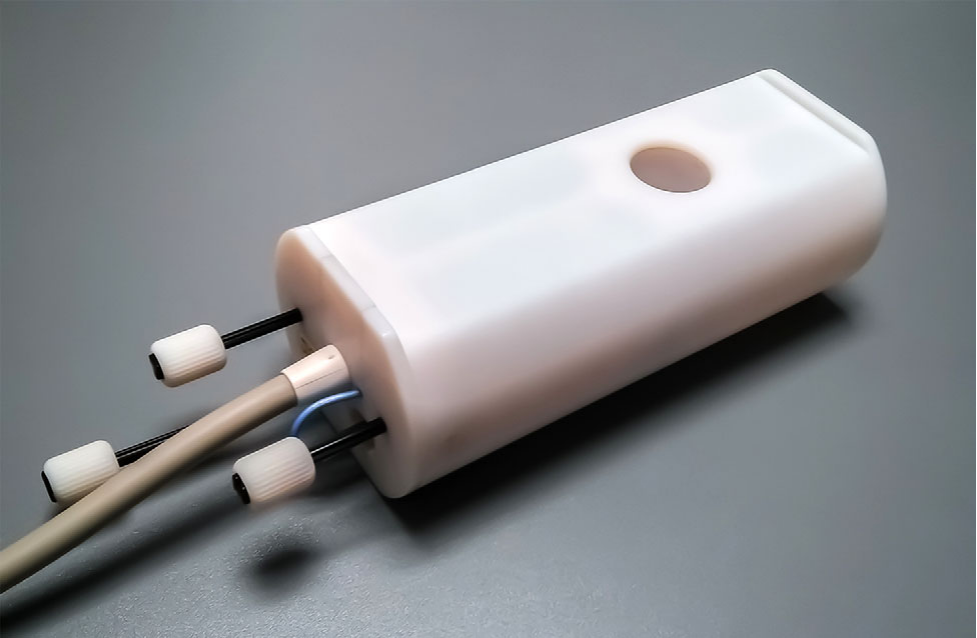
Photograph of the final implementation of the finger coil in its 3D‐printed housing. Rods for manual tuning and matching, as well as a longer rod for fixation in a micro‐gradient bore, are visible on the bottom left side.

The solenoid was made of 5‐mm‐wide copper strips wound on cylindrical polytetrafluoroethylene substrate. The coil housing was fabricated using a 3D‐printer (Objet Eden 350, Stratasys, Eden Prairie, MN, USA), which accommodates the coil, transmit–receive switch, and low‐noise preamplifier, as well as mechanical parts for manual tuning and matching (Fig. 1).

#### Bench Measurements

To assess coil efficiency, the quality factor Q (Q‐factor) was measured in unloaded and loaded configuration on a network analyzer (E5061B, Agilent, Santa Clara, USA) using the −3 dB bandwidth method. For the loaded condition, in vivo human fingers were inserted in the measurement volume of the coil.

#### Subjects

The phalanx lengths and maximum finger circumferences on all fingers of the right hand, as well as the Q‐factor of the coil loaded by the respective right index finger, were measured on 10 healthy volunteers (5 female, 5 male; aged 32 ± 7 years). MRI was performed on the right index finger of six healthy volunteers (2 female, 4 male; aged 28.5 ± 4.9 years). The finger was wrapped with a 5‐mm foam pad before being inserted in the bore of the solenoid coil to keep it in position without exerting too much pressure. Care was taken to position the subjects as comfortably as possible in the superman position. Arms and hands were fixated with sand cushions to limit motion. The study was conducted after informed written consent was given, and in accordance with the Declaration of Helsinki.

### MR Imaging

#### MR Scanner

A 7T whole‐body MRI system (Magnetom 7T, Siemens Healthineers, Erlangen, Germany) equipped with an SC72d gradient coil (maximum gradient strength 70 mT/m, slew rate 200 T/m/s) was used for MR measurements.

#### MR Imaging Sequences

Three different MR imaging sequences (see Table 1) were adapted to the specific demands of high‐resolution finger imaging in clinically feasible measurement times. MR sequences 1 and 2 were 2D TSE sequences using an echo train length of 7. To speed up the measurement, MR sequence 1 was adapted for highest in‐plane resolution in a cross‐section of the finger. MR sequence 2 was oriented in coronal slices (i.e., parallel to the plane of the flat hand) with the aim to visualize bone and cartilage structures. MR sequence 3 is a 3D MPRAGE sequence, employing an initial 180 ° inversion pulse followed by a gradient echo imaging sequence. This sequence provides good contrast for a variety of soft tissues present in the finger. To further improve their visibility, the fat signal was suppressed by selective water excitation.

**Table 1:** MR‐Sequence Parameters

	Sequence	TF	TR (ms)	TI (ms)	TE (ms)	Matrix	FOV (mm^2^)	Resolution (µm)	No. of Slices	Slice Thickness (µm)	BW (Hz/px)	Fat Suppression	TA (min)
1	2D TSE axial	7	7450	–	26	464 × 348	27 × 27	58 × 78	60	500	101	no	6:06
2	2D TSE coronal	7	4570	–	37	960 × 288	96 × 28	100 × 97	43	500	260	no	3:08
3	3D MPRAGE	–	2730	1700	6.71	512 × 256	100 × 50	195 × 195	128	200	130	yes	8:45

BW, read‐out bandwidth per pixel; FOV, field of view; MPRAGE, magnetization prepared rapid acquisition gradient echo; TA, acquisition time; TE, echo time; TF, turbo factor (echo train length); TI, inversion time; TR, repetition time; TSE, turbo spin echo.

#### Image Postprocessing

MRI data were explored using the image viewing and reconstruction software available on the MR scanner console (SyngoVB17, Siemens Healthineers, Erlangen, Germany). The curved slice reconstruction included in that software was used to obtain nonplanar slices aligned with interesting anatomical features, such as the flexor tendons, central line of the bones, vascular structures, or nail bed.

From the isotropic data of MR‐sequence 3, selected anatomical features were manually segmented using MRIcro (www.mricro.com). Separate volumes of interest (VOI) were drawn for bone, blood vessels, nerves, Pacinian corpuscles, flexor tendons, extensor tendons, and annular ligaments. Signal‐to‐noise and contrast‐to‐noise ratios were calculated for these tissue types. The VOI data were imported in 3D Slicer (www.slicer.org) and smoothed using Gaussian filters with a full width at half maximum of 0.1 mm (annular ligaments), 0.2 mm (vessels, nerves, extensor tendons), or 0.4 mm (bones, flexor tendons). Triangulated surface models of the respective structures were obtained using a threshold value of 0.1 (annular ligaments) or 0.3 (all other structures). The volumetric MRI data were thresholded and displayed with an opacity gradient to provide a contour reference for the 3D renderings. Each VOI was assigned a specific color as a labeling.

## RESULTS

### Radiofrequency Coil

#### Design and Simulation

The optimal values obtained for parameters *α* and *β* were *α* = *β* = 80 °, resulting in a more homogeneous B_1_ field than for a regular solenoid (Supporting Figs. S1b,c). The B_1_ intensity dropoff along the central axis at the edges of the coil was reduced from 45% of the central value in a regular solenoid to 25% for the optimized geometry (Supporting Fig. S1d).

Three‐dimensional electromagnetic simulation resulted in a maximum 10 g‐averaged SAR value of 40 W/kg per Watt of incident power, assuming lossless conductors. The coil is defined as a local volume transmit coil on the MR scanner, obeying the 20 W/kg local SAR limit for extremities in the normal operating mode defined in the International Electrotechnical Commission guidelines 17.

#### Bench Measurements

The unloaded Q‐factor was 209 ± 0.4 and the loaded Q‐factor was 106 ± 10. The resulting Q‐factor ratio is 2.0 ± 0.2; this means that the coil is operated at the border between the sample noise‐ and the coil noise‐dominated regime. This can be because the finger contains few conductive tissues (i.e., no muscles but mostly fat, bone, and connective tissues), leading to relatively low sample losses.

#### Imaging Results

**Figure 2:**
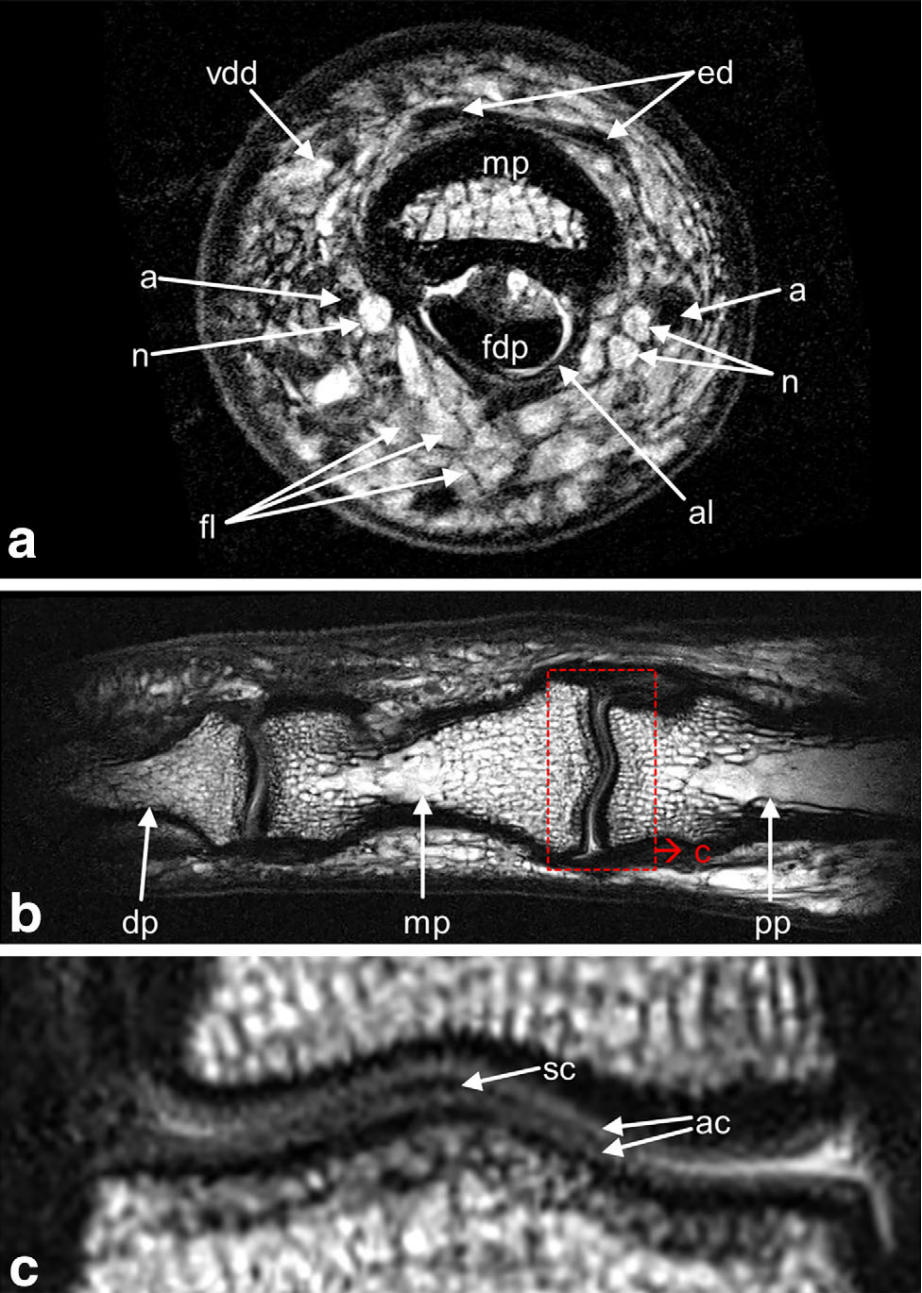
Finger cross‐section, bones and cartilage. (**a**) MR‐sequence 1: 2D TSE, 78 × 58 µm^2^ in‐plane resolution, slice thickness 500 µm, acquisition time 6 min 6 s. (**b**,**c**) MR sequence 2: 2D TSE, 97 × 100 µm^2^ in‐plane resolution, 500 µm slice thickness, acquisition time 3 min 8 s. (**b**) The distal and proximal interphalangeal joints are displayed on a curved slice along the central axis of the bones. (**c**) Zoomed detail of the proximal interphalangeal joint, corresponding to the red box in (b). a, artery (left: arteria volaris indicis radialis; right: arteria digitalis palmaris propria); ac, articular cartilage; al, annular ligament A4; dp, distal phalanx; ed, extensor digitorum tendon; fdp, flexor digitorum profundus tendon; fl, fat lobules; mp, middle phalanx; n, nerve; pp, proximal phalanx; sc, synovial cavity; vdd, vena digitalis dorsalis.

An ultrahigh resolution cross‐section of the finger in the center of the middle phalanx acquired with MR sequence 1 is shown in Figure 2a. At an in‐plane resolution of 78 × 58 µm^2^, it features the two strands of the flexor digitorum profundus and the extensor digitorum tendons, the thin hypointense sheet of the annular ligament A4, the bone marrow of the middle phalanx, the index finger branches of the radial and ulnar arteries, both digital nerves, fat lobules, the hypointense dermis, and the hyperintense epidermis layer.

For detailed visualization of the bone structure and the cartilage layers of the interphalangeal joints, the curved slice reconstruction along the central axis of the phalanxes from MR sequence 2 is shown in Figures 2b,c. The thin layers of articular cartilage and the synovial cavity can be distinguished.

**Figure 3:**
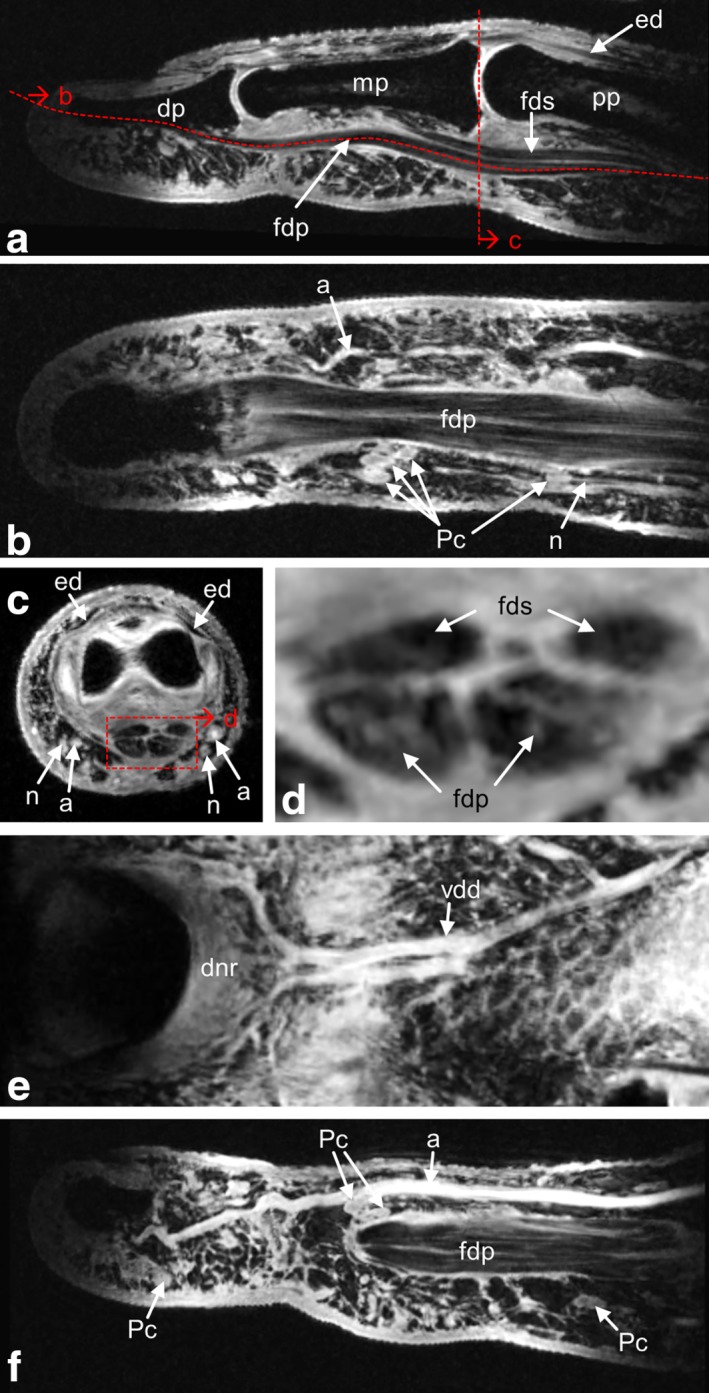
Finger tendons (**a‐d**) and vasculature (**e, f**). MR sequence 3: 3D magnetization‐prepared rapid acquisition gradient echo with fat saturation, 195 × 195 × 200 µm^3^ resolution, acquisition time 8 min 45 s. (**a**) Sagittal slice along the flexor digitorum tendons. (**b**) Curved, quasi‐coronal slice along the flexor digitorum superficialis tendons. Clustered Pacinian corpuscles along the digital nerve are clearly observed; arteria digitalis palmaris propria also is visible. (**c**) Axial slice at the position of the proximal interphalangeal joint. (**d**) Zoomed detail of the flexor digitorum tendon cross‐section marked by the red box in (c). (**e**) Curved slice reconstruction along the curvature of the nail bed visualizes the branching of the vena digitalis dorsalis and the dorsal nail roof. (**f**) Curved slice reconstruction along arteria digitalis palmaris propria. Some Pacinian corpuscles can be identified. a, artery; dnr, dorsal nail roof; dp, distal phalanx; ed, extensor digitorum tendon; fdp, flexor digitorum profundus tendon; fds, flexor digitorum superficialis tendon; mp, middle phalanx; pp, proximal phalanx; n, nerve; Pc, Pacinian corpuscles; vdd, vena digitalis dorsalis.

From MR sequence 3, reconstructions highlighting tendons (Figs. 3a‐d) and vascular structures (Figs. 3e,f) were derived. In Figure 3b, a curved slice along the flexor digitorum profundus tendon reveals the internal structure of the tendon 18, as well as cross‐sections of Pacinian corpuscles alongside the digital nerve. Blood vessels are shown in Figures 3e,f, in which Figure 3e represents a slice curved along the nail bed to show the branching of vena digitalis dorsalis and the dorsal nail roof, and Figure 3f represents a slice curved along the path of arteria digitalis palmaris propria. Again, Pacinian corpuscles, and also a part of the flexor tendon are visible.

**Figure 4:**
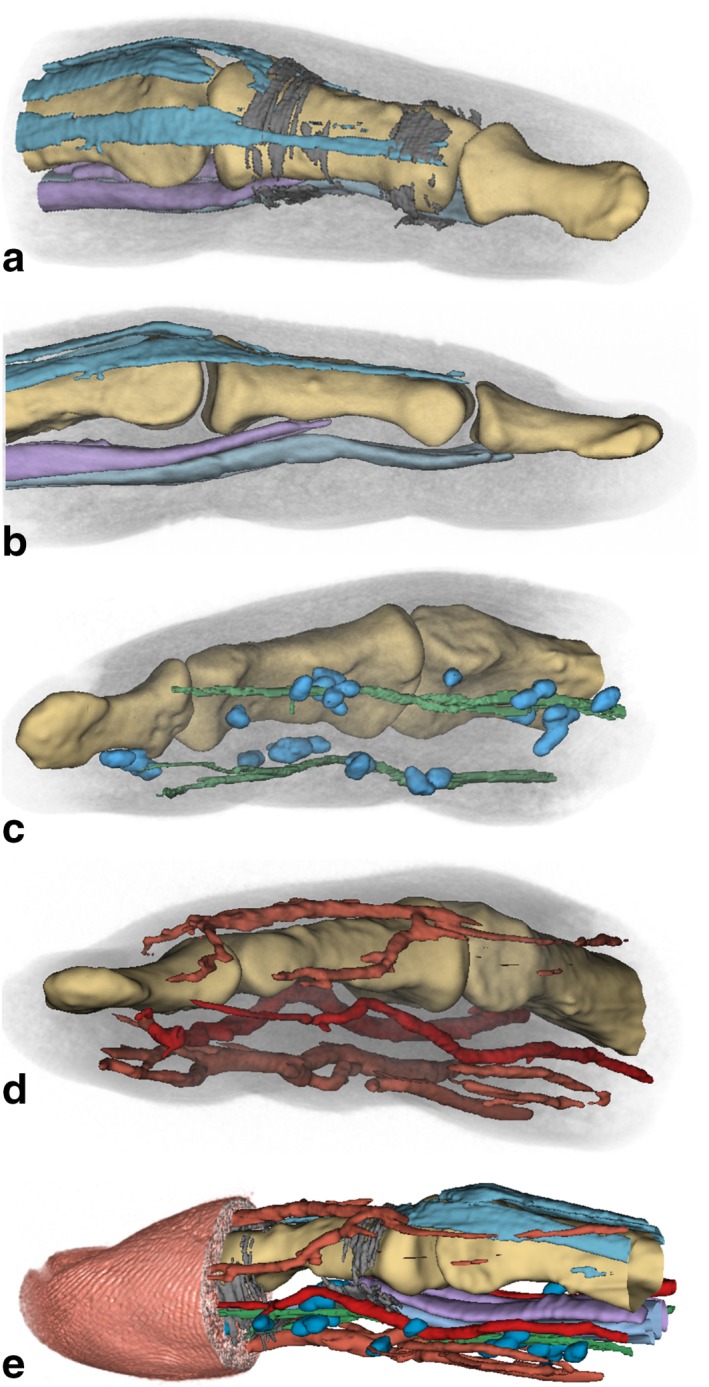
3D visualizations of finger structures. MR sequence 3: manually segmented VOIs. (**a**) Bones (yellow), extensor digitorum tendons (turquoise), and annular ligaments (dark gray) A4 (left) and A5 (right). (**b**) Extensor digitorum tendons (turquoise) and flexor digitorum profundus (light blue) and superficialis (purple) tendons. (**c**) Nerves (green) and Pacinian corpuscules (blue) arranged in clusters along the nerves. (**d**) Arteria volaris indicis radialis and arteria digitalis palmaris propria (dark red), vena digitalis dorsalis, and vena digitalis palmaris (light red). (**e**) Skin surface reconstruction (skin color) with the fingerprints visible. All segmented structures depicted in (a) to (d) are shown together.

Figure 4 shows 3D renderings from segmented data of the 200‐µm isotropic MR sequence 3 for bones, tendons, and ligaments (Figs. 4a,b); bones, nerves, and Pacinian corpuscles (Fig. 4c); bones and blood vessels (Fig. 4d); and all abovementioned structures, together with a surface rendering of the fingertip with the finger prints clearly visible (Fig. 4e). The shape and position of tendons can be readily observed. Annular ligaments, however, are only partly visible due to their small thickness and partial volume effects, mainly where their orientation is at an angle of around 45 ° with respect to the voxel edges. Pacinian corpuscles are arranged in grape‐like clusters along the digital nerves, which in turn are closely positioned along the arteries, matching the findings of anatomical cadaver 19 and light microscopy studies 20. As expected from textbook anatomy, digital nerves and arteries are located laterally, whereas larger veins are found on the dorsal and palmar sides of the finger.

Signal‐to‐noise and contrast‐to‐noise ratio values for the segmented structures are reported in Supporting Table S2.

## DISCUSSION

The results show that high‐resolution in vivo finger MRI is feasible, with excellent image quality in patient‐compatible measurement times below 10 min through combination of dedicated radiofrequency (RF) hardware, ultrahigh magnetic field strength, well‐adapted acquisition sequences, and sophisticated image postprocessing.

Imaging at very high spatial resolution usually requires low readout bandwidth, resulting in considerable chemical shift between water and fat, making it advantageous to employ fat‐suppression techniques—especially in fingers, where subcutaneous fat constitutes a large portion of the total volume.

Visualizing data sets in 3D enables following individual structures in context to each other and might help to identify pathologies more easily. Currently, the anatomy of the finger is too complex for automatic segmentation algorithms, requiring manual segmentation of large data sets.

## CONCLUSION

For complete 3D visualization of the finger ligaments, the achieved spatial resolution of 200 µm isotropic is not yet sufficient; however, improved hardware, together with adapted sequence design, could be a solution in future studies.

Such hardware improvement could be achieved by using a loop–gap resonator instead of a solenoid, and by using it as a transmit‐only coil. Combined with an array of miniaturized surface coils 21 for signal reception, this would increase sensitivity and enable parallel imaging techniques to alleviate constraints in echo time or shorten the total acquisition time.

Potential clinical applications comprise musculoskeletal investigations already established for larger body structures, such as the knee or ankle 22, 23, but require even higher spatial resolution when applied to the finger.

## Supporting information

Additional supporting information may be found in the online version of this article

**Fig. S1**. RF coil design and optimization. a) The helical structure with variable slope parameter is shown exemplarily for α = β = 180˚. The segments with zero, linearly changing, and constant pitch are marked by arrows. b) Simulated $B_{1}^{+}$ field distribution for the optimized geometry (left) and a regularly wound solenoid (right) as a reference. c) Histogram of the $B_{1}^{+}$ field in the inner coil volume showing the improvement in homogeneity of the optimized solenoid (red) versus the regular solenoid (green). The overlap of the red and green areas is shown in a brownish color. d) Line plot of the $B_{1}^{+}$ field amplitude along the central axis of the solenoid. It can be seen that the drop‐off at the edges is reduced from ∼45% to ∼25% of the central value for the optimized solenoid.
**Table S2**. Signal‐to‐noise ratios and Contrast‐to‐noise ratios for MR sequence 3.
