# Supplementary material for: In vivo MRI of the human finger at 7 T
Source: Magn Reson Med. 2017 Mar 10;79(1):588–92. doi: 10.1002/mrm.26645 (PMC5763334; doi:10.1002/mrm.26645)
Supplement: Supplementary file 1 — Fig. S1. RF coil design and optimization. a) The helical structure with variable slope parameter is shown exemplarily for α = β = 180˚. The segments with zero, linearly changing, and constant pitch are marked by arrows. b) Simulated B1+ field distribution for the optimized geometry (left) and a regularly wound solenoid (right) as a reference. c) Histogram of the B1+ field in the inner coil volume showing the improvement in homogeneity of the optimized solenoid (red) versus the regular solenoid (green). The overlap of the red and green areas is shown in a brownish color. d) Line plot of the B1+ field amplitude along the central axis of the solenoid. It can be seen that the drop‐off at the edges is reduced from ∼45% to ∼25% of the central value for the optimized solenoid. Table S2. Signal‐to‐noise ratios and Contrast‐to‐noise ratios for MR sequence 3. [file MRM-79-588-s001.pdf]

Supporting Figure S1.

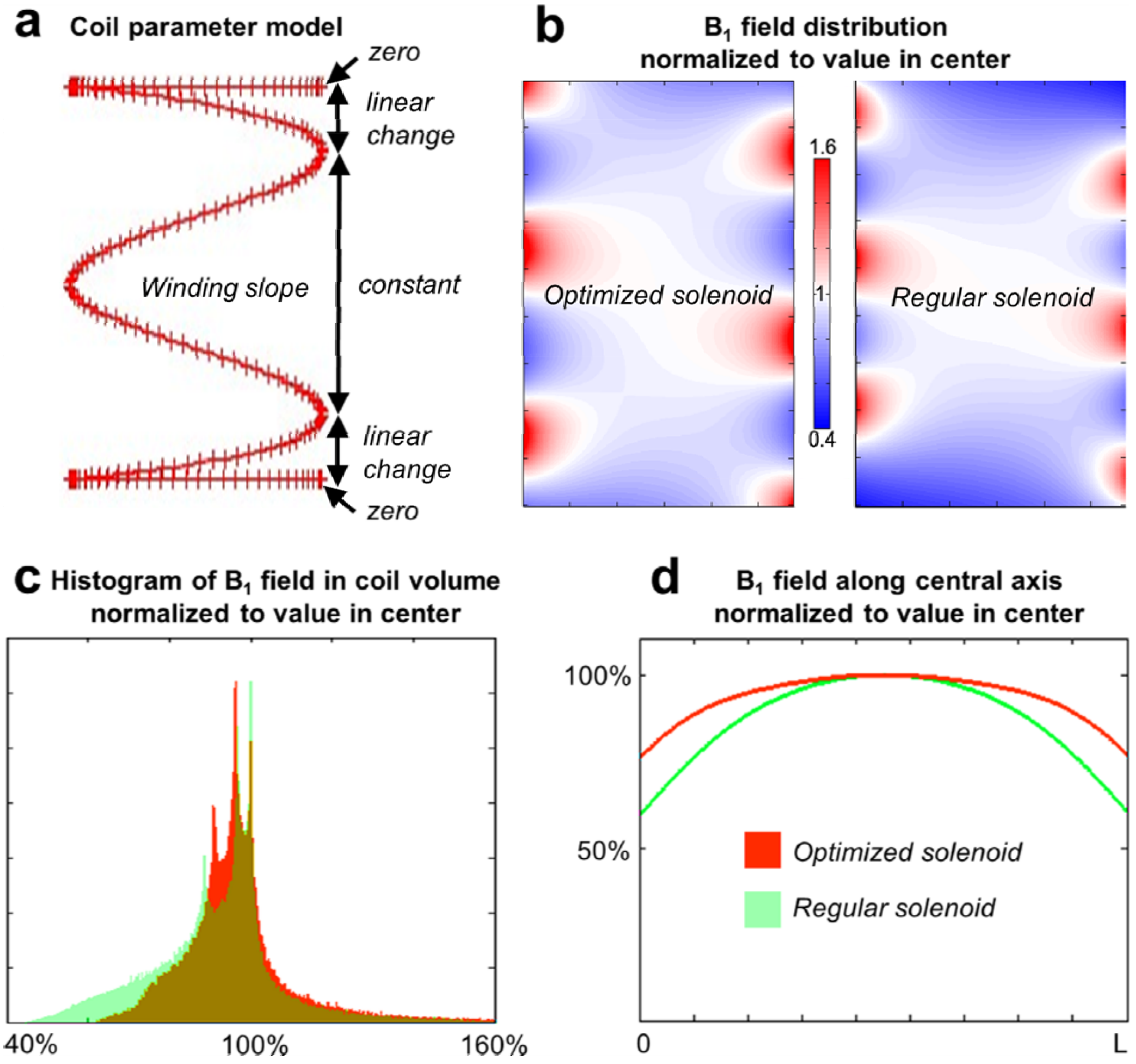

RF coil design and optimization.

a) The helical structure with variable slope parameter is shown exemplarily for $\alpha = \beta = 180^{\circ}$. The segments with zero, linearly changing, and constant winding slope are marked by arrows.

b) Simulated B_1 field distribution for the optimized geometry (left) and a regularly wound solenoid (right) as a reference.

c) Histogram of the B_1 field in the inner coil volume showing the improvement in homogeneity of the optimized solenoid (red) versus the regular solenoid (green). The overlap of the red and green areas is shown in a brownish color.

d) Line plot of the B_1 field amplitude along the central axis of the solenoid. It can be seen that the drop-off at the edges is reduced from ~45% to ~25% of the central value for the optimized solenoid.

Supporting Table S2.

Signal-to-noise ratios and Contrast-to-noise ratios for MR sequence #3.

MR sequence #3	SNR	CNR			
		Arteries	Veins	Vater-Pacinian Corpuscules	Nerves
**Tendons**	11	31	29	18	21
**Nerves**	29	12	10	3	
**Vater-Pacinian Corpuscules**	33	9	7		
**Veins**	40	2			
**Arteries**	42				
